# Upregulated Interleukin 21 Receptor Enhances Proliferation and Epithelial-Mesenchymal Transition Process in Benign Prostatic Hyperplasia

**DOI:** 10.3389/fendo.2019.00004

**Published:** 2019-01-23

**Authors:** Deqiang Xu, Ping Chen, He Xiao, Xinghuan Wang, Michael E. DiSanto, Xinhua Zhang

**Affiliations:** ^1^Department of Urology, Zhongnan Hospital of Wuhan University, Wuhan, China; ^2^Department of Surgery and Biomedical Sciences, Cooper Medical School of Rowan University, Camden, NJ, United States

**Keywords:** benign prostatic hyperplasia, prostatitis, inflammation, interleukin 21 receptor, cell apoptosis, cell cycle, epithelial-mesenchymal transition

## Abstract

**Background:** Interleukins (ILs) and related chronic inflammation have been found to contribute to the development of benign prostatic hyperplasia (BPH) in recent decades. As a late member of the ILs family, IL-21 receptor (IL-21R) can modulate cell proliferation, however, IL-21R activity in the prostate has not been examined. The current study aimed to elucidate a potential role of IL-21R in the development of BPH.

**Material and Methods:** Human prostate tissues, cell lines and rats were used. QRT-PCR, Western blot, and immunohistochemistry, along with hematoxylin and eosin, Masson's trichrome, and immunofluorescent staining were performed. BPH-1 cells with IL-21R silenced were cultured or co-cultured with macrophages (active THP-1, AcTHP-1). Apoptosis and cell cycle phases were determined via flow cytometry. Epithelial-mesenchymal transition (EMT) processes were also examined. *In vivo*, rat prostatitis was induced with intraprostatic injected lipopolysaccharide (LPS).

**Results:** IL-21R was highly expressed in human as well as rat prostate, mainly in the epithelial compartment. BPH concomitant with prostatitis significantly upregulated the expression of IL-21R. Knockdown of IL-21R induced cell apoptosis and cycle arrest at G0/G1 phase, and blocked the EMT process in BPH-1 cells. When IL-21R silenced BPH-1 cells were co-cultured with AcTHP-1 cells, these aforementioned processes and IL-21R change were completely reversed. Prostatic hyperplasia was observed with IL-21R upregulated in LPS induced prostatitis rats. More specifically, the expression of apoptosis, cyclin, and EMT proteins in this rat model are altered in a manner consistent with that seen in the cell line model.

**Conclusions:** Our novel data demonstrates the expression and functional activities of IL-21R in the mechanism for development of BPH. IL-21R mainly localized in prostate epithelium and it was upregulated in hyperplastic prostate tissues. IL-21R enhanced proliferation of BPH-1 cells, via inhibiting cell apoptosis, and modulating cell cycles, as well as the EMT process, in response to inflammatory stimuli.

## Introduction

Benign prostatic hyperplasia (BPH) is a common urinary tract disease in aging men (1), resulting in lower urinary tract symptoms (2). The incidence is close to 50% among men over the age of 50 and 80% among men over the age of 80 (3). Age and androgens are considered necessary for the pathogenesis of BPH (4), while the exact etiology of BPH remains unclear. In recent decades, many hypotheses have been proposed suggesting that androgens-to-estrogens ratio imbalance, stromal-epithelial interactions, growth factors, and metabolic syndrome including obesity and diabetes, likely play important roles in the onset and progression of BPH. Recent evidence suggested that BPH development involves accumulation of mesenchymal-like cells derived from the prostatic epithelium by epithelial-mesenchymal transition (EMT) (5–7). Meanwhile, it has been also hypothesized that BPH is an immune-mediated inflammatory disease and chronic inflammation may directly stimulate the development of BPH (8–10). Clinically, most BPH patients often concomitantly have prostatitis (10, 11). Inflammatory infiltrates of BPH tissues may lead to tissue damage, a chronic process of wound healing, tissue remodeling, and prostate growth (12, 13). Histologically, there are often many infiltrating lymphocytes and macrophages around the glandular elements of BPH tissues (14). These infiltrating cells produce cytokines to stimulate epithelial and stromal proliferation (15). Indeed, Robert et al. (16) found that macrophages are one of the major inflammatory infiltrates in BPH specimens as determined by histological analysis, suggesting macrophages are crucial for promoting BPH development and progression via secretion of inflammatory factors. Meanwhile, profound EMT features were observed in an LPS-induced prostatitis and BPH rat model (17).

Interleukins (ILs) are the main lymphatic cytokines with multiple biological activities and have important regulatory roles in the development, differentiation, and immune response of immune cells as well as the activation of other cells. In recent decades, IL-21 and its receptor IL-21R were identified and newly added to the ILs system (18, 19), which shares a similar structure to the other members of this family (20). Binding of IL-21 to the IL-21R activates the Janus-faced kinase/signal transducer and activator of transcription (JAK/STAT) pathways and plays important roles in enhancing cell proliferation (21). IL-21R is preliminarily expressed on hematopoietic cells and regulates the proliferation of mature B and T cells in response to stimuli (19). However, studies have shown IL-21R is also expressed on non-immune cells such as fibroblasts, endothelial cells, keratinocytes, and Hodgkin lymphoma cells (22, 23). A recent study showed IL-21 and its receptor play an important role in breast cancer cells via proliferation, migration, and invasion (20), suggesting IL-21R could contribute to other diseases via similar mechanisms. Since BPH occurs due to the imbalance of cell proliferation and cell apoptosis, as well as immune-mediated chronic inflammation as aformentioned, IL-21 and its receptor IL-21R could play a permissive role in the development of BPH. Indeed, elevated levels of ILs have been found in BPH tissues when compared to normal prostate tissues including IL-1, IL-6, and IL-8 (24, 25). Overexpression of IL-15/IL-15R in the prostate has been found in BPH patients when compared to their controls (26). Moreover, recent studies have suggested that both prostatic epithelial and stromal cells can express ILs, including IL-1, IL-2, IL-4, IL-6, IL-8, IL-12, IL-13, IL-15, IL-17, IL-18, and IL-23, in response to inflammatory stimuli. Subsequently these ILs were found to regulate the growth of epithelial and stromal cells (15, 27), indicating ILs play important roles in the onset and progression of BPH. Although the production of these ILs result in the abnormal growth in the prostate, the primary initiators during the development of BPH are not yet known.

Nevertheless, the expression and functions of IL-21R in the prostate have never been determined. In the current study, we examined the expression and functional activities of IL-21R in prostate tissues and human prostate cell lines. We hypothesized that IL-21R would respond to inflammatory stimuli from macrophages and play an important role in the onset and progression of BPH, via inhibiting apoptosis, modulation of cell cycle progression and the EMT process, which would provide a new molecular target for BPH therapy.

## Materials and Methods

### Animals and Tissues

A total of 16 male Sprague–Dawley rats (12 weeks old) were used and randomly divided into two groups. Rats were anesthetized with pentobarbital sodium, and then the lower abdomen was incised to expose the prostates. Subsequently, PBS (200 μl) or LPS (200 μg/kg, Sigma, St. Louis, MO, USA) (17) was injected equally into the right and left prostate lobes in PBS (control; *n* = 8) and LPS groups (*n* = 8), respectively. On the 14th day after injection, rat prostates were excised, weighed, and used for the following experiments. Fifteen prostate samples from young brain-dead men (mean age, 28.2 ± 4.4 years old) undergoing organ donation were obtained as controls and 15 BPH samples were obtained from patients (mean age, 69.4 ± 5.7 years old) undergoing cystoprostatectomy for infiltrating bladder cancer without prostate infiltration. Post-operative prostate pathology examinations revealed BPH concomitant with chronic prostatitis. All human samples were obtained after the approval of the Hospital Committee for Investigation in Humans and after receiving written informed consent from all patients or their relatives. Prostate tissues were divided into two strips and were, respectively, stored in liquid nitrogen for PCR analysis and Western blotting analysis and stored in 10% neutral buffered formalin for histological examination and immunofluorescence microscopy. All animal protocols were approved by the Animal Experiment Center of Zhongnan Hospital of Wuhan University and human studies were conducted in accordance with the principles of the Declaration of Helsinki.

### Cell Culture

Human benign prostatic enlargement epithelia cell line BPH-1 (Cat. #BNCC339850) was purchased from the Procell Co., Ltd. in Wuhan, China. Identification of the cell lines was performed at the China Center for Type Culture Collection in Wuhan, China. SV40 large-T antigen-immortalized stromal cell line WPMY-1 (Cat. #GNHu36) was purchased from the Stem Cell Bank, Chinese Academy of Sciences in Shanghai, China. Human acute monocytic leukemia cell line THP-1 (SCSP-567) was obtained from Stem Cell Library of Chinese Academy of Sciences. The BPH-1 cells were cultured in RPMI-1640 medium (Gibco, China) containing 10% fetal bovine serum (FBS) (Gibco, Australia). The WPMY-1 cells were cultured in DMEM medium (Gibco, China) containing 1% penicillin G sodium/streptomycin sulfate and 5% FBS. The THP-1 cells were cultured in Opti medium with 10% inactivated FBS, the THP-1 cells were differentiated into macrophages (active THP-1, AcTHP-1) using 10 ng/ml LPS for 24 h. All the cell lines were cultured in a humidified atmosphere consisting of 95% air and 5% CO_2_ at 37°C.

### SiRNA and Transfection

The cells were transiently transfected with siRNA using Lipofectamine transfection reagent. When the BPH-1 cells were 30–50% confluent in six-well culture plates, the cell culture medium was replaced with fresh RPMI-1640 medium 30 min before transfection. The transfection media were prepared according to the manufacturer's instructions and incubated at room temperature for 10 min. Subsequently, 200 μl of the lipofectamine complex solution was added to each well. After incubation for 6 h at 37°C in 5% CO_2_, the cell culture medium was replaced with fresh RPMI-1640 medium and incubated for 48 h. The GFP fluorescence was evaluated as a reporter for the transfection efficiency. The sequence of each siRNA is summarized in Supplementary Table S1.

### Co-culture Experiments

Six-well transwell plates (Corning Inc., Corning, NY, USA) were used for co-culture experiments. THP-1 cells (1 × 10^6^ cells) were differentiated into AcTHP-1 in the upper insert (0.4 μm) of six-well transwell plates containing 500 μl of Opti medium with 10 ng/ml LPS for 24 h. BPH-1 cells (3 × 10^5^ cells) were seeded in the lower chamber of six-well transwell plates containing 1.5 ml of RPMI-1640 with 10% FBS medium. The two cell lines were co-cultured for 48 h in a humidified atmosphere consisting of 95% air and 5% CO_2_ at 37°C.

### Flow Cytometry Analysis

For cell cycle analysis, BPH-1 cells (1 × 10^6^ cells) were harvested, washed with PBS, and then centrifuged. Pellets were resuspended with 1 ml DNA staining solution, which contained 50 μg/ml propidium iodide and 0.1 mg/ml RNaseA, and 10 μl permeabilization solution. The DNA content distribution was analyzed by flow cytometry analysis (Beckman, Cat. #FC500) after incubation in the dark at 37°C for 30 min. For cell apoptosis analysis, FITC Annexin V Apoptosis Detection Kit I (BD Biosciences, USA) was used. BPH-1 cells (1 × 10^6^ cells) were harvested and then stained with FITC Annexin V Apoptosis Detection Kit I according to the manufacturer's instruction.

### MTT Assays

Three thousand BPH-1 cells were plated in each well of 96-well plates. Exactly 20 μl of 5 mg/ml MTT solution was added to each well on day 0, 1, 2, 3 and 4, and the cells were incubated for 4 h at 37°C in an incubator. The media was then removed and 150 μl of DMSO was added. The plates, covered with tinfoil, were shaken on an orbital shaker for 10 min. The readings were recorded by a microplate reader (Cat. #SpectraMax M2, Molecular Devices, Sunnyvale, CA, USA) at an absorbance of 490 nm and reported as relative cell proliferation values.

### RNA Extraction and Quantitative Real-Time PCR (qRT-PCR) Analysis

Total RNA was isolated from frozen tissues and cell lines using Trizol reagent (Invitrogen, Carlsbad, CA, USA) according to the manufacturer's instructions and quantitated at 260/280 nm using a NanoPhotometer spectrophotometer (IMPLEN, Westlake Village, CA, USA). Two μg of total RNA was reverse-transcribed to complementary DNA (cDNA) via the SuperScript II First-Strand Synthesis System according to the manufacturer (Invitrogen). QRT-PCR was performed to determine the level of mRNA expression of a gene of interest based on SYBR green using a Bio-Rad (Hercules, CA, USA) CFX96 system. The expression levels of genes were normalized to the expression of GAPDH mRNA and compared by 2^−ΔΔ*CT*^ method. Primer sequences are listed in Table 1. All samples were independently repeated for analysis three times.

**Table 1:** Primer sequence used for qPCR.

**Target gene**	**Primer sequence**
**HUMAN**	
**IL-21R**	
Forward	5′-GGCAAGACCAGTATGAAGAGC-3′
Reverse	5′-TGACACTGAAAATGTCGTCGG-3′
**GAPDH**	
Forward	5′-ATCCCATCACCATCTTCCAGGAG-3′
Reverse	5′-CCTGCTTCACCACCTTCTTGATG-3′
**RAT**	
**IL-21R**	
Forward	5′-GACCTGGAGTGAGTGGAGTG-3′
Reverse	5′-TAGCCTCCAAGGCAGATGGT-3′
**GAPDH**	
Forward	5′-ACAGCAACAGGGTGGTGGAC-3′
Reverse	5′-TTTGAGGGTGCAGCGAACTT-3′

### Western Blot Analysis

Tissues and BPH-1 cells were lysed and ultrasonicated in RIPA reagent containing protease inhibitor and phosphatase inhibitor (Sigma-Aldrich) on ice for 30 min. After centrifugation at 12,000 × g for 15 min, supernatant was collected. Thirty μg of total protein was separated via a 10% sodium dodecyl sulfate-polyacrylamide gel (SDS-PAGE) and transferred to polyvinylidene fluoride membrane (Millipore, Billerica, MA, USA) using a Bio-Rad wet transfer system. The membranes were blocked in TBST (Tris-buffered saline with 0.05% Tween 20) containing 5% non-fat dry milk at room temperature for 2 h and then incubated with primary antibodies (listed in Supplementary Table S2) overnight at 4°C. After washing for three to five times with TBST, the membranes were incubated with secondary antibody (listed in Supplementary Table S3) at room temperature for 2 h. Detection of reaction antigen was performed with an enhanced chemiluminescence kit (Thermo Scientific Fisher, Waltham, MA, USA). The bands were quantified by Quantity One® 1-D Analysis software (Bio-Rad). The expression levels of protein were normalized to the expression of GAPDH. All samples were independently repeated three times and means determined.

### Hematoxylin and Eosin (H & E) Staining

Prostate paraffin sections (5 μm) were deparaffinized in xylene for 3 × 10 min, rehydrated in descending concentrations of ethanol (100%, 96%, 80%, 70%) and H_2_- O. The sections were then stained in 10% Hematoxilin (Sigma-Aldrich) for 7 min, followed by washing under the tap water for 10 min to reveal the nuclei. Afterwards, the sections were stained in 1% Eosin (Sigma-Aldrich) containing 0.2% glacial acetic acid for 5 min. After staining, the sections were washed with tap water, dehydrated in increasing grades of ethanol (70%, 80%, 96%, 100%), and cleared in xylene for 3 × 10 min. The sections were imaged by an inverted phase contrast microscope (Cat. #DMI 1, Leica, Wetzlar, Germany).

### Masson's Trichrome Staining

As previously described (28), prostate tissues were embedded into paraffin after being fixed in 10% formalin for 24–36 h and cut into 5 μm sections. Then, the sections were stained using Masson's trichrome staining. Staining was detected by light microscopy. Prostatic smooth muscle (SM) cells, collagen fibers, and epithelial cells were stained red, blue, and orange, respectively. In each sample, we analyzed three areas randomly under magnification (× 200). The area percentage of SM, collagen fibers, and glandular epithelium were quantitated with Image Pro Plus 5.0.

### Immunohistochemistry Staining

Tissues were embedded into paraffin after being fixed in 10% formalin for 24–36 h and cut into 5 μm sections. The sections were then deparaffinized in xylene and rehydrated using descending grades of alcohols (100%, 95%, 70%, 30%). Antigen retrieval was performed by heating the sections in 10 mM sodium citrate buffer at pH 6.0 and at 96°C for 30 min. Endogenous peroxidase activity was blocked using PBS containing 3% hydrogen peroxide for 15 min. Non-specific binding was blocked by incubating the sections in 5% normal donkey serum with 2% BSA for 1 h. The sections were then incubated overnight at 4°C with rabbit anti-IL-21R antibodies (1:100; Abcam) followed by incubation with secondary antibodies at room temperature for 1 h. Horse radish peroxidase polymer conjugate (Invitrogen, Carlsbad, CA, USA) was used to localize the antibody bound to antigen, with diaminobenzidine as the final chromogen. All immunostained sections were lightly counterstained with hematoxylin and immunostaining was detected by light microscopy.

### Immunofluorescent Staining

Tissues were frozen and sectioned in 5 μm thick slices and thaw-mounted onto glass slides using a cryostat (Leica CM 1850, Wetzlar, Germany), air-dried, and fixed for 10 min in ice cold acetone. BPH-1 cells (6 × 10^5^ cells) treated as described above were re-cultured on glass slides in six-well culture plates for 24 h, washed with PBS three times and fixed with 4% formaldehyde at room temperature for 30 min. Tissue and cell slides were then washed in PBS and incubated for 2 h in a mixture of PBS supplemented with 0.2% Triton X-100 and 0.1% bovine serum albumin. After incubation overnight with the monoclonal antibodies rabbit anti-IL-21R (1:100; Abcam, Cambridge, MA), rabbit anti-E-cadherin (anti-E-Cad), anti-N-cadherin (anti-N-Cad), and anti-vimentin (all 1:100; all Bioworld Technology, Atlanta, GA, USA) at 4°C, slides were incubated with FITC-labeled secondary antibody (1:1,000; Santa Cruz Biotechnology, Santa Cruz, CA) for 1 h at room temperature. DAPI was used for staining the nucleus. Stained tissues and cells were viewed by fluorescence microscopy (PerkinElmer Life Sciences, Akron, OH, USA).

### Statistical Analysis

The data values were expressed as the means ± standard deviation (*SD*). Statistical analysis was performed using non-parametric tests and one-way analysis of variance (ANOVA). Difference was considered statistically significant at *P* < 0.05.

## Results

**Figure 1:**
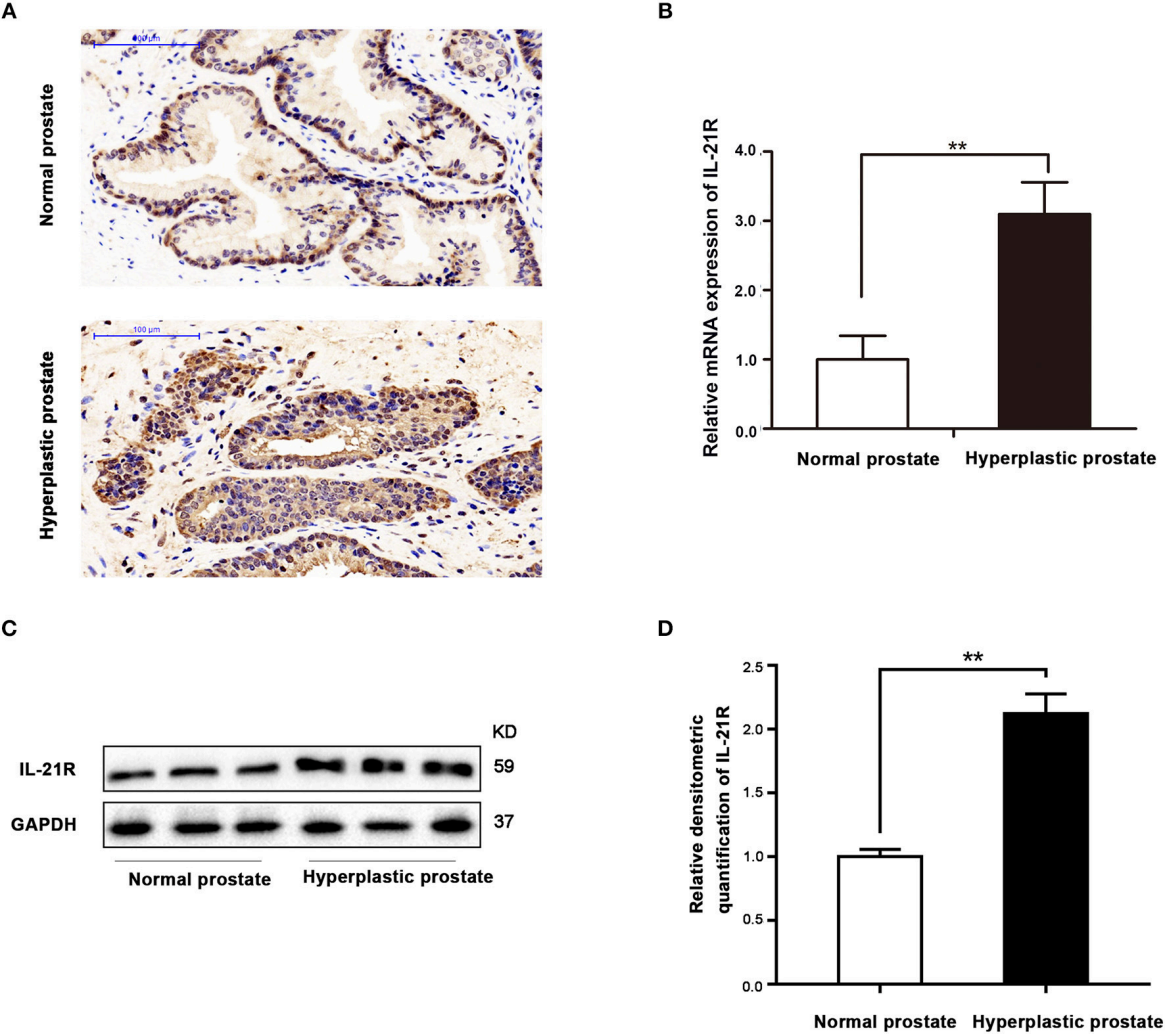
Localization and expression of IL-21R in human prostate. **(A)** Immunohistochemistry of IL-21R in human prostate. Upper, Normal prostate; Lower, Hyperplastic prostate. IL-21R mainly distributed in epithelium. The scale bars are 100 μm; **(B)** the mRNA expression of IL-21R in human prostate (*n* = 15 for each group). Bars, ± SD; ^**^P < 0.01 vs. normal prostate. **(C)** Representative Western Blot band of IL-21R in human prostate. **(D)** Relative densitometric quantification of IL-21R in human prostate. GAPDH expression was analyzed as a loading control, results are expressed as ratio of IL-21R in respect to GAPDH. Boxes, mean; bars, ± SD; ^**^P < 0.01 vs. normal prostate.

Immunohistochemical staining found IL-21R was predominantly localized in the epithelial compartment of human prostate with slight staining observed in the stroma (Figure 1A). Moreover, IL-21R mRNA and protein are barely detectable with qRT-PCR and Western Blot in cultured stromal cells (Supplementary Figure S1). Therefore, epithelial cells were used in our subsequent studies. In addition, IL-21R expression was higher in human hyperplastic prostate tissues (Figure 1A) than normal prostate tissues, which was further determined by qRT-PCR and Western Blot. As shown in Figures 1B–D, both mRNA and protein expression of IL-21R were significantly increased (*P* < 0.05) in hyperplastic prostate by 3.1- and 2.1-fold, respectively.

**Figure 2:**
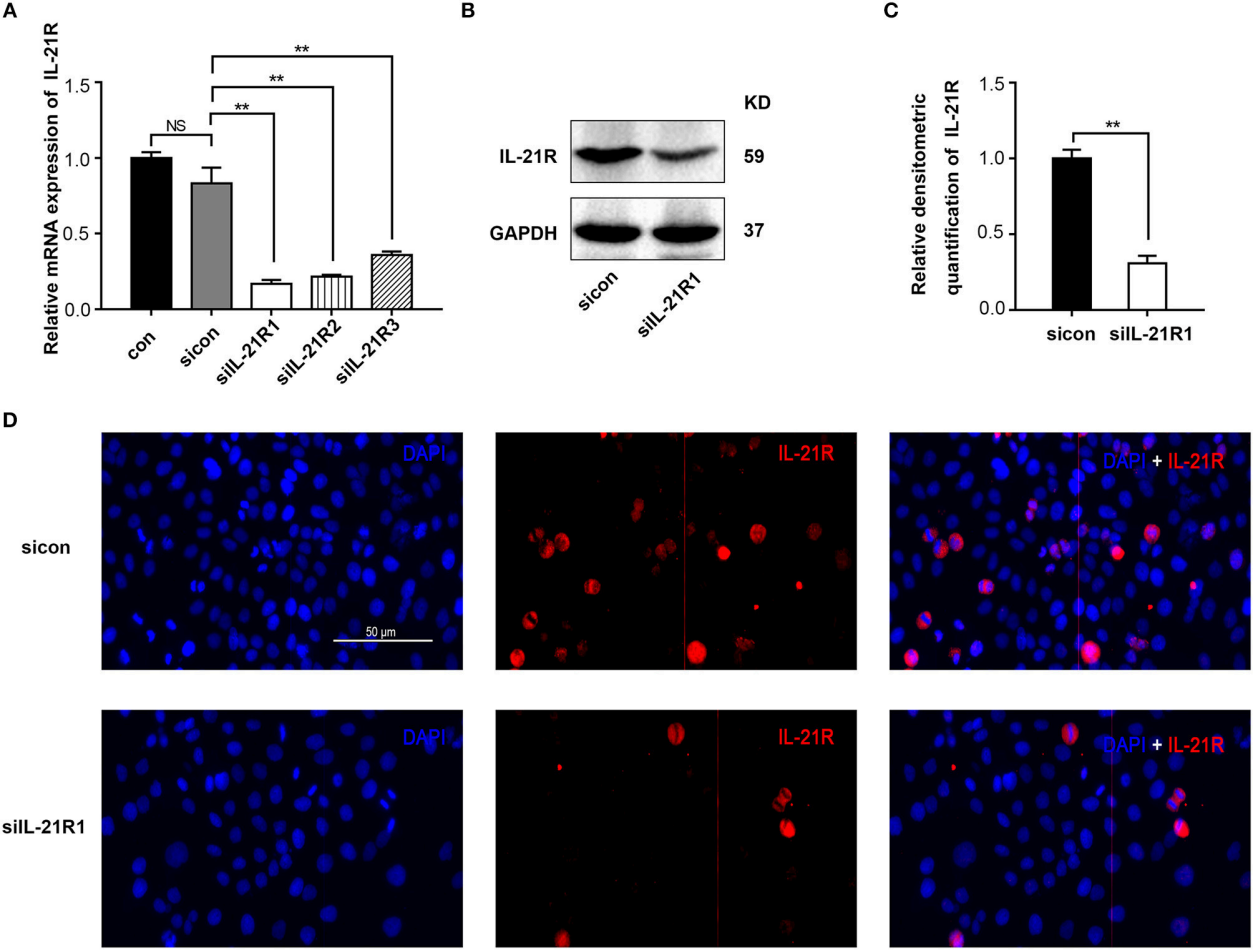
Knockdown of IL-21R in BPH-1 cells with siRNA. **(A)** Knockdown efficiency of IL-21R in the mRNA levels in BPH-1 cells with three different siRNA sequences (siIL-21R 1, 2, and 3). Boxes, mean; bars, ± SD; con = control; NS means no significance, ^**^P < 0.01 vs. control-siRNA (sicon). **(B)** Representative Western Blot band of IL-21R in BPH-1 cells treated with siIL-21R of the highest inhibitory efficiency (siIL-21R1) and sicon. **(C)** Relative densitometric quantification of IL-21R in BPH-1 cells. GAPDH expression was analyzed as a loading control, results are expressed as ratio of IL-21R in respect to GAPDH. Boxes, mean; bars, ± SD; ^**^P < 0.01 vs. sicon. **(D)** Immunofluorescence of IL-21R in BPH-1 cells treated with siIL-21R1 and sicon. Cy3-immunofluorescence (red) indicates IL-21R expression. DAPI (blue) indicates cell nuclear staining. Merged image indicates of IL-21R and DAPI. The scale bars are 100 μm.

**Figure 3:**
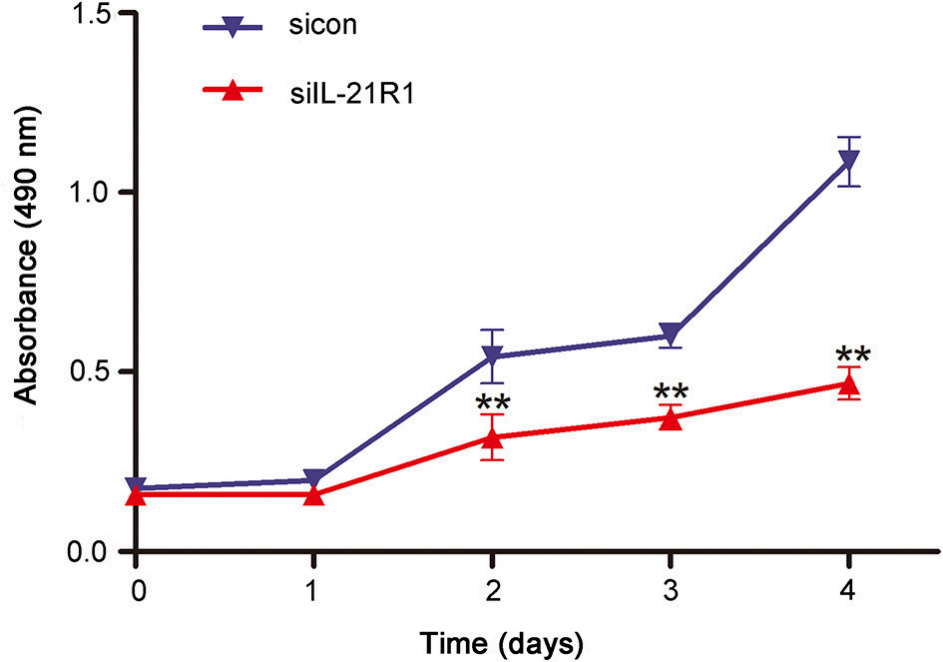
Effect of knockdown of IL-21R on BPH-1 cell proliferation. Viability of BPH-1 cells treated with siIL-21R1 and sicon from day 0 until day 4 was analyzed by MTT assays. MTT assays was performed at the absorbance at 490 nm. The X axis indicates times, the Y axis indicates the absorbance of BPH-1 cells at 490 nm. Bars, ± SD; ^**^P < 0.01 vs. sicon.

**Figure 4:**
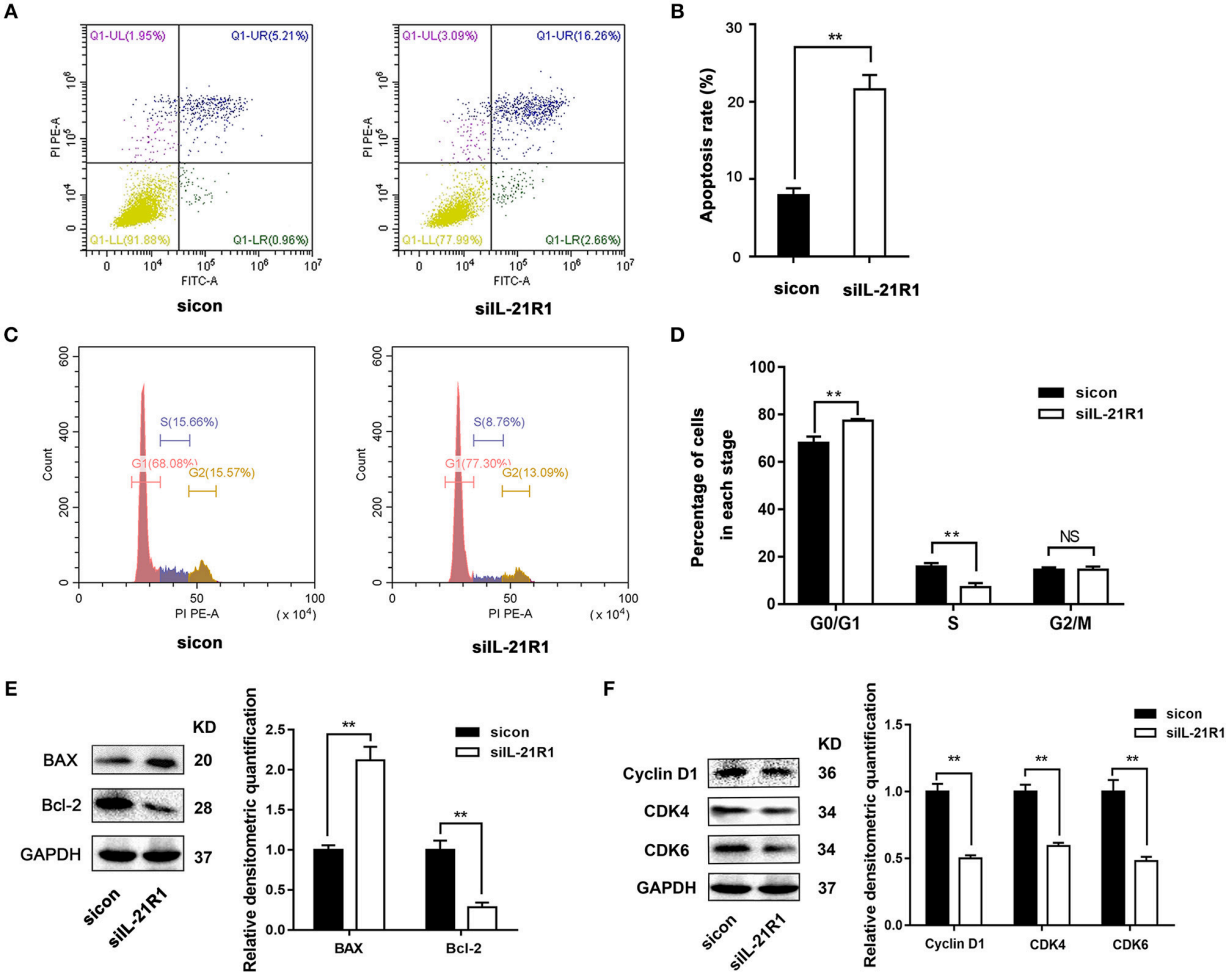
Effect of knockdown of IL-21R on BPH-1 cell apoptosis and cell cycle. **(A)** Flow Cytometry analyses for cell apoptosis in BPH-1 cells treated with siIL-21R1 and sicon. **(B)** Bar graph for apoptosis rate of BPH-1 cells. Boxes, mean; bars, ± SD; ^**^P < 0.01 vs. sicon. **(C)** Flow Cytometry analyses for cell cycle in BPH-1 cells treated with siIL-21R1 and sicon. **(D)** Bar graph for the percentage of BPH-1 cells in each stage. Boxes, mean; bars, ± SD; ^**^P < 0.01 vs. sicon. **(E)** Left, Representative Western Blot band of cell apoptosis associated proteins (BAX and Bcl-2) in BPH-1 cells. Right, Relative densitometric quantification of cell apoptosis associated protein (BAX and Bcl-2) in BPH-1 cells. GAPDH expression was analyzed as a loading control, results are expressed as ratio of the proteins in respect to GAPDH. Boxes, mean; bars, ± SD; ^**^P < 0.01 vs. sicon. **(F)** Left, Representative Western Blot band of cell cycle associated protein (Cyclin D1, CDK4, and CDK6) in BPH-1 cells. Right, Relative densitometric quantification of cell cycle associated protein (Cyclin D1, CDK4, and CDK6) in BPH-1 cells. GAPDH expression was analyzed as a loading control, results are expressed as ratio of the proteins in respect to GAPDH. Boxes, mean; bars, ± SD; ^**^P < 0.01 vs. sicon.

**Figure 5:**
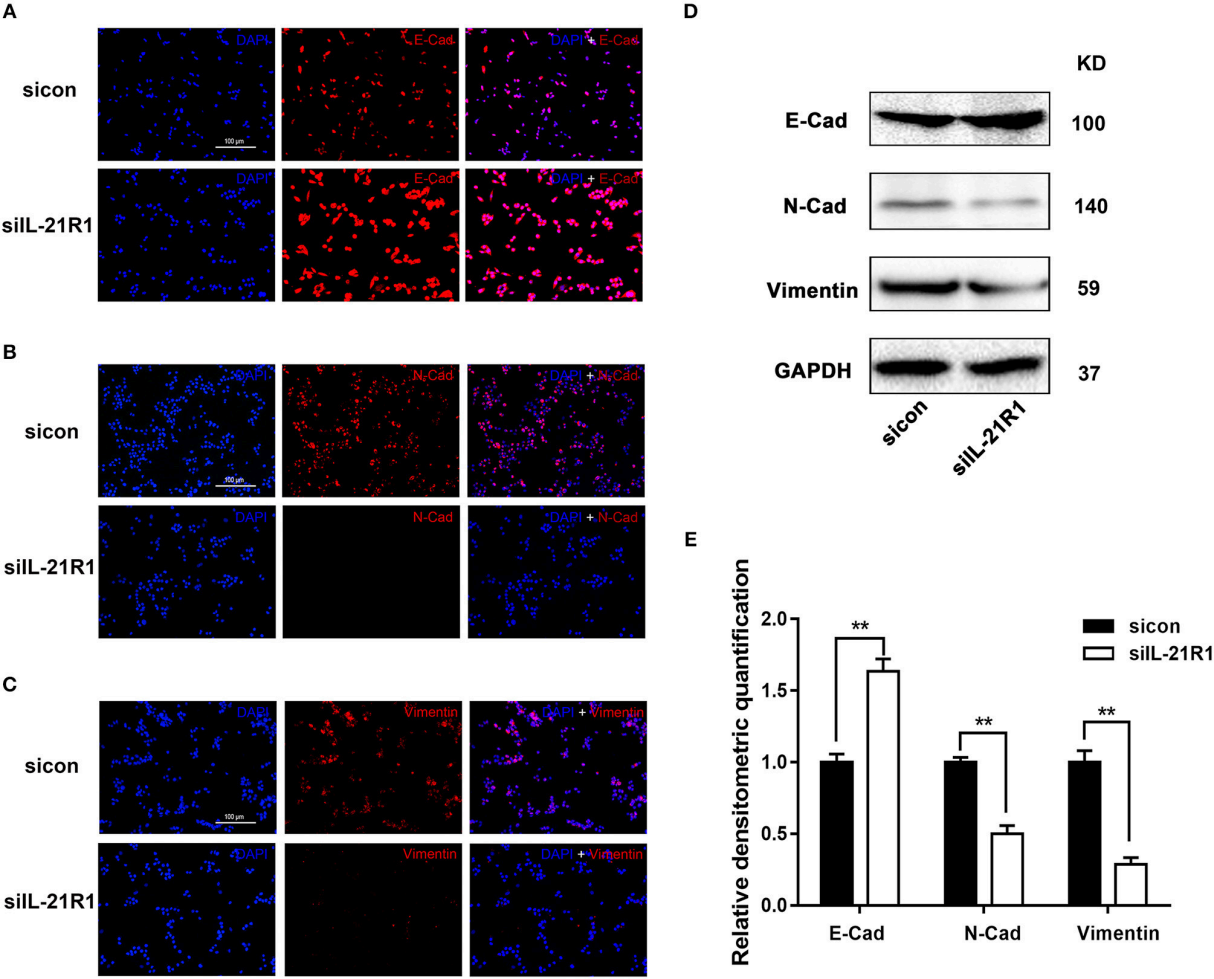
Effect of knockdown of IL-21R on EMT process in BPH-1 cells. **(A–C)** Immunofluorescence of E-Cad, N-Cad, and vimentin in BPH-1 cells treated with siIL-21R1 and sicon. Left, DAPI (blue) indicates cell nuclear staining. Middle, Cy3-immunofluorescence (red) indicates E-Cad **(A)**, N-Cad **(B)**, and vimentin **(C)** expression. Right, Merged image. The scale bars for **(A–C)** are 100 μm. **(D)** Representative Western Blot bands of EMT associated protein (E-Cad, N-Cad, and vimentin) in BPH-1 cells. **(E)** Relative densitometric quantification of EMT associated proteins (E-Cad, N-Cad, and vimentin) in BPH-1 cells. GAPDH expression was analyzed as a loading control, results are expressed as ratio of the proteins in respect to GAPDH. Boxes, mean; bars, ± SD; ^**^P < 0.01 vs. sicon.

As aforementioned, the epithelial cell line BPH-1 was employed to evaluate the role of IL-21R in the development of BPH. We knocked down IL-21R expression in BPH-1 cells by using three different siRNA sequences (siIL-21R 1, 2, and 3) and the mRNA expression of IL-21R was down-regulated (Figure 2A) by 80, 74, and 57%, respectively. siIL-21R1 was selected for use in further experimentation due to its highest inhibitory efficacy. Indeed, the expression of IL-21R in BPH-1 cells transfected with siIL-21R1 was obviously decreased both in Western Blot study (Figures 2B,C) and immunofluorescent staining (Figure 2D). To investigate the functions of IL-21R in prostate epithelial cells, we further analyzed cell proliferation, cell apoptosis, and cell cycle progression for these IL-21R down-regulated BPH-1 cells, using MTT assay and flow cytometry analysis. MTT assay showed siIL-21R1 treated BPH-1 cells grew slower than control group (Figure 3). Flow Cytometry showed an increase of cell apoptosis by 12.8% (Figures 4A,B). Flow Cytometry also showed an increase of G0/G1 phase cells and a decrease of S phase cells by 9.2 and 6.9%, respectively (Figures 4C,D). Meanwhile, we detected the levels of proteins associated with cell apoptosis and the cell cycle, using Western Blot. BAX expression was increased while Bcl-2 expression was decreased (Figure 4E) by 2.1-folds and 72%, respectively. Cyclin D1, CDK4, and CDK6 were all significantly decreased (Figure 4F). Furthermore, to determine whether differential expression of IL-21R altered the EMT process of BPH-1 cells, we detected the expression of proteins (E-Cad, N-Cad, and vimentin) associated with EMT by Western blot and immunofluorescent staining. As shown in Figure 5, E-Cad was up-regulated while N-Cad and vimentin were down-regulated. The results indicated that knockdown of IL-21R could inhibit the EMT process. Our data suggests that knockdown of IL-21R could significantly inhibit the proliferation of prostatic epithelium via triggering cell apoptosis and arresting the cell cycle, as well as attenuation of the EMT process.

**Figure 6:**
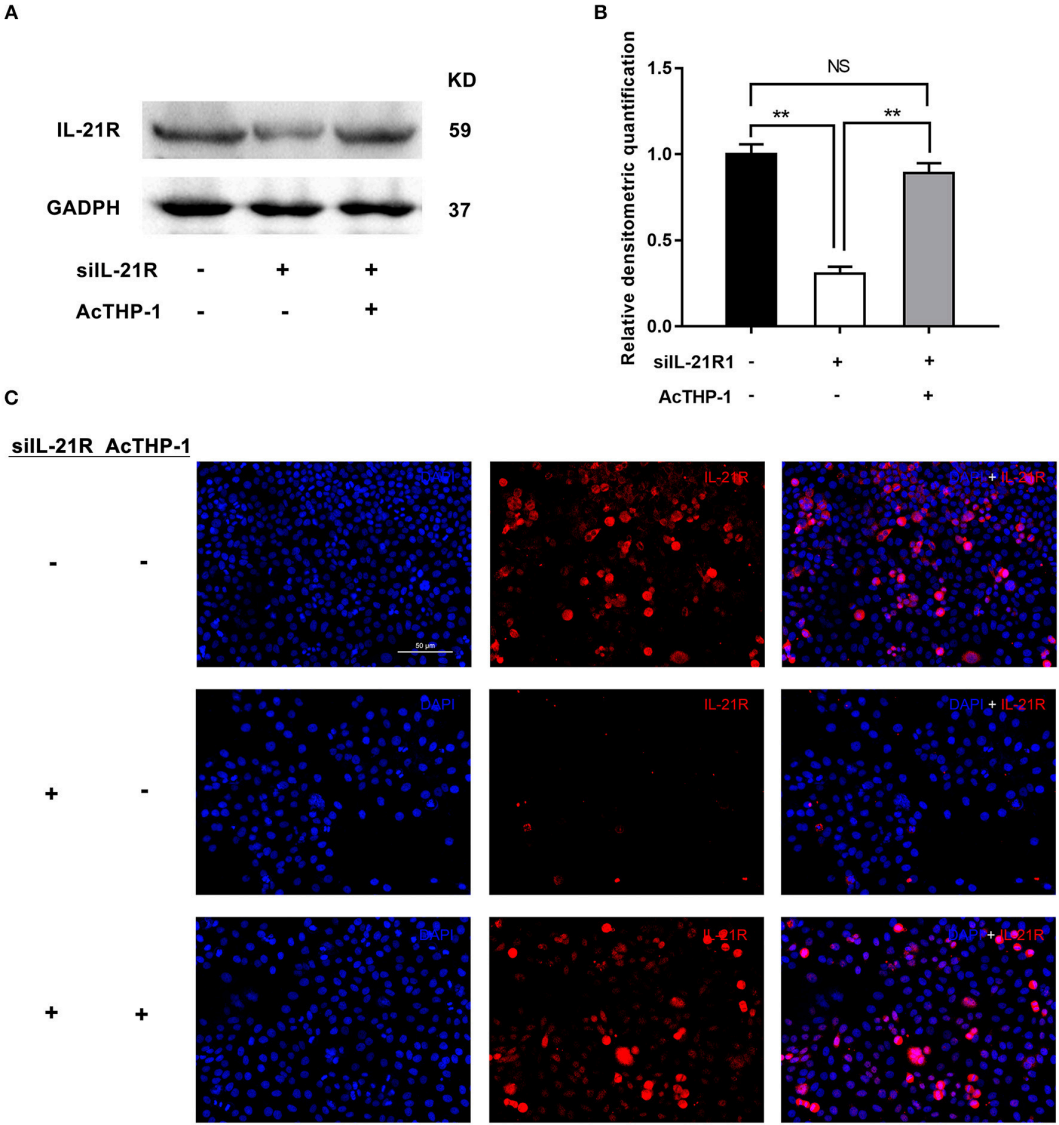
Effect of AcTHP-1 co-culture on the expression of IL-21R in BPH-1 cells. **(A)** Representative Western Blot bands of IL-21R in BPH-1 cells co-cultured with or without AcTHP-1 in the absence or presence of siIL-21R1. **(B)** Relative densitometric quantification of IL-21R in BPH-1 cells. GAPDH expression was analyzed as a loading control, results are expressed as ratio of the proteins in respect to GAPDH. Boxes, mean; bars, ± SD; ^**^P < 0.01 vs. BPH-1cells without AcTHP-1 co-culture in the presence of siIL-21R1. NS means no significance, BPH-1 cells without AcTHP-1 co-culture in the absence of siIL-21R1 vs. BPH-1cells with AcTHP-1 co-culture in the presence of siIL-21R1. **(C)** Immunofluorescence of IL-21R in BPH-1 cells. Cy3-immunofluorescence (red) indicates IL-21R expression. DAPI (blue) indicates cell nuclear staining. Merged image indicates of IL-21R and DAPI. Upper, BPH-1cells without AcTHP-1 co-culture in the absence of siIL-21R1. Middle, BPH-1cells without AcTHP-1 co-culture in the presence of siIL-21R1. Lower, BPH-1cells with AcTHP-1 co-culture in the presence of siIL-21R1. The scale bars are 50 μm.

**Figure 7:**
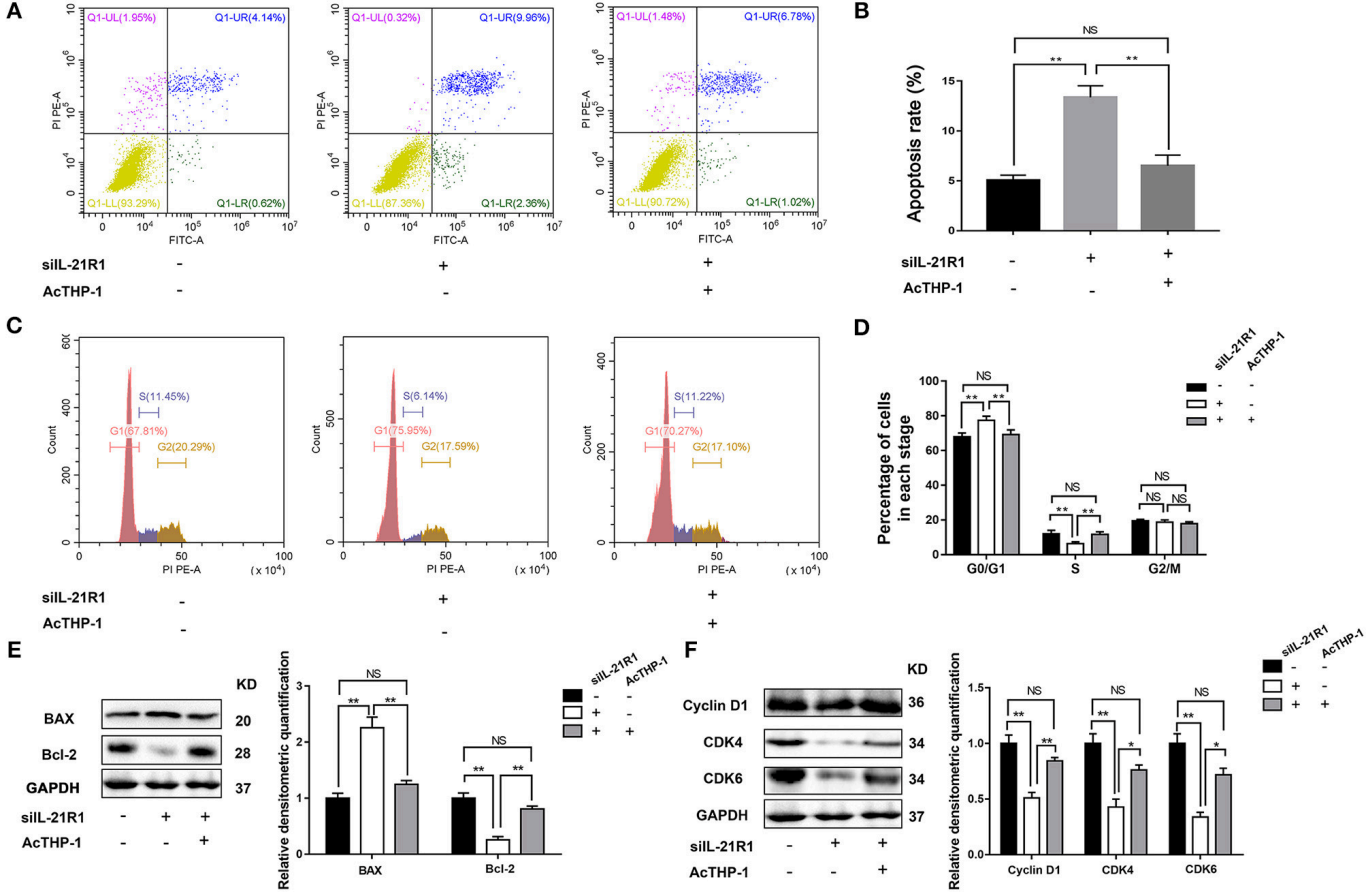
Effect of AcTHP-1 co-culture on BPH-1 cell apoptosis and cell cycle. **(A)** Flow Cytometry analyses for cell apoptosis in BPH-1 cells co-cultured with or without AcTHP-1 in the absence or presence of siIL-21R1. **(B)** Bar graph for the rate of BPH-1 cell apoptosis. Boxes, mean; bars, ± SD; ^**^P < 0.01 vs. BPH-1cells without AcTHP-1 co-culture in the presence of siIL-21R1. NS means no significance, BPH-1 cells without AcTHP-1 co-culture in the absence of siIL-21R1 vs. BPH-1cells with AcTHP-1 co-culture in the presence of siIL-21R1. **(C)** Flow Cytometry analyses for cell cycle in BPH-1 cells co-cultured with or without AcTHP-1 in the absence or presence of siIL-21R1. **(D)** Bar graph for the percentage of BPH-1 cells in each cell phase. Boxes, mean; bars, ± SD; ^**^P < 0.01 vs. BPH-1 cells without AcTHP-1 co-culture in the presence of siIL-21R1. NS means no significance, BPH-1 cells without AcTHP-1 co-culture in the absence of siIL-21R1 vs. BPH-1cells with AcTHP-1 co-culture in the presence of siIL-21R1. **(E)** Left, Representative Western Blot band of cell apoptosis associated protein (BAX and Bcl-2) in BPH-1 cells. Right, Relative densitometric quantification of cell apoptosis associated protein (BAX and Bcl-2) in BPH-1 cells. GAPDH expression was analyzed as a loading control, results are expressed as ratio of the proteins in respect to GAPDH. Boxes, mean; bars, ± SD; ^**^P < 0.01 vs. BPH-1cells without AcTHP-1 co-culture in the presence of siIL-21R1. NS means no significance, BPH-1cells without AcTHP-1 co-culture in the absence of siIL-21R1 vs. BPH-1 cells with AcTHP-1 co-culture in the presence of siIL-21R1. **(F)** Left, Representative Western Blot band of cell cycle associated protein (Cyclin D1, CDK4, and CDK6) in BPH-1 cells. Right, Relative densitometric quantification of cell cycle associated protein (Cyclin D1, CDK4, and CDK6) in BPH-1 cells. GAPDH expression was analyzed as a loading control, results are expressed as ratio of the proteins in respect to GAPDH. Boxes, mean; bars, ± SD; ^*^P < 0.05, ^**^P < 0.01 vs. BPH-1 cells without AcTHP-1 co-culture in the presence of siIL-21R1. NS means no significance, BPH-1cells without AcTHP-1 co-culture in the absence of siIL-21R1 vs. BPH-1cells with AcTHP-1 co-culture in the presence of siIL-21R1.

**Figure 8:**
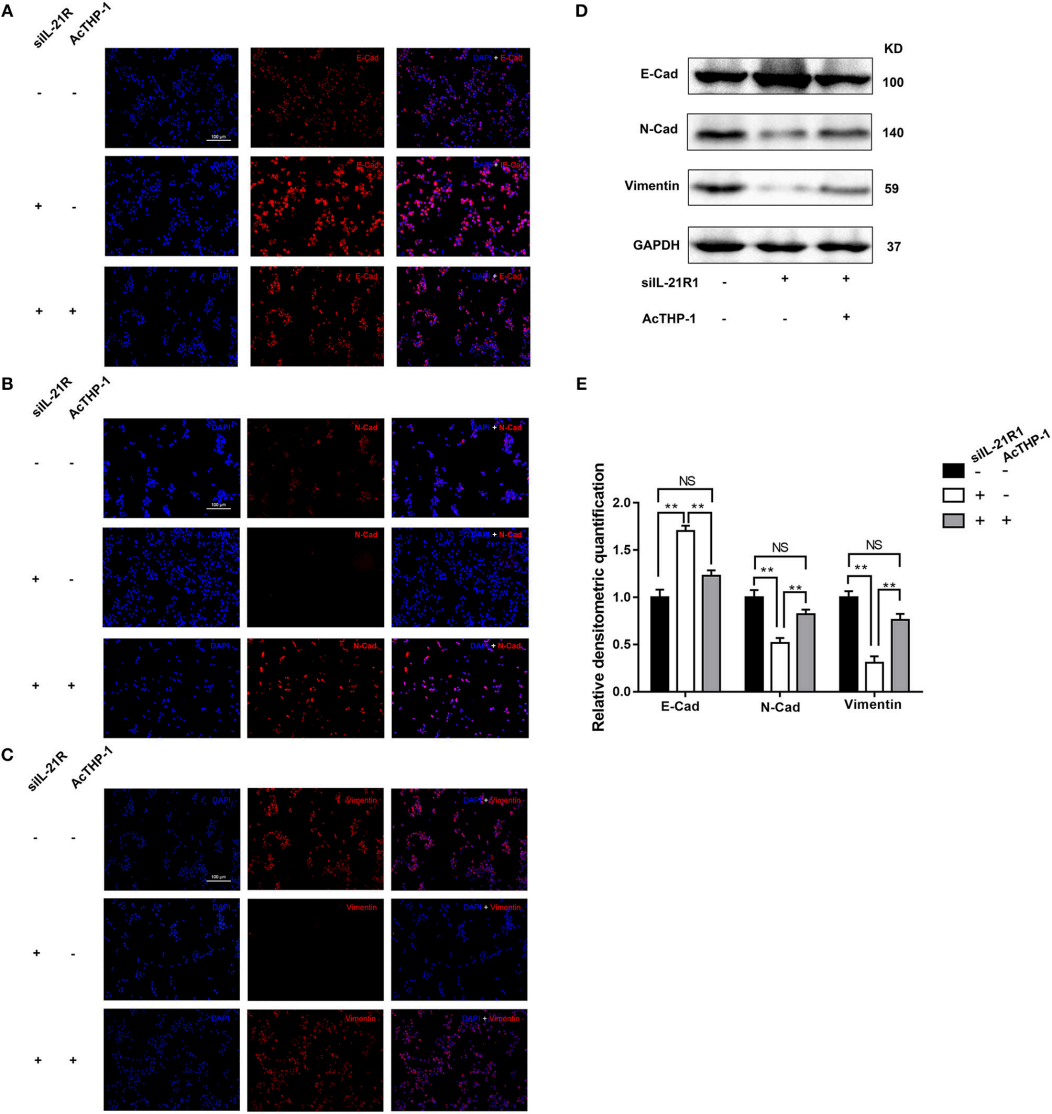
Effect of AcTHP-1 co-culture on EMT process in BPH-1 cells. **(A–C)** Immunofluorescence of E-Cad **(A)**, N-Cad, **(B)** and vimentin **(C)** in BPH-1 cells co-cultured with or without AcTHP-1 in the absence or presence of siIL-21R1. Left, DAPI (blue) indicates cell nuclear staining. Middle, Cy3-immunofluorescence (red) indicates E-Cad **(A)**, N-Cad **(B)**, and vimentin **(C)** expression. Right, Merged image. The scale bars for **(A–C)** are 100 μm. **(D)** Representative Western Blot bands of EMT associated protein (E-Cad, N-Cad, and vimentin) in BPH-1 cells. **(E)** Relative densitometric quantification of EMT associated protein (E-Cad, N-Cad, and vimentin) in BPH-1 cells. GAPDH expression was analyzed as a loading control, results are expressed as ratio of the proteins in respect to GAPDH. Boxes, mean; bars, ± SD; ^**^P < 0.01 vs. BPH-1cells without AcTHP-1 co-culture in the presence of siIL-21R1. NS means no significance, BPH-1cells without AcTHP-1 co-culture in the absence of siIL-21R1 vs. BPH-1 cells with AcTHP-1 co-culture in the presence of siIL-21R1.

To further determine the activity of IL-21R in the prostatic epithelial cells, we co-cultured the above transfected BPH-1 cells with or without AcTHP-1. LPS was used for differentiation of THP-1 cells into macrophages. To exclude the effect of THP-1 cells or LPS on BPH-1 cells, we co-cultured THP-1 cells with BPH-1 cells or stimulated BPH-1 cells with gradient concentration of LPS. Then, we determined the expression of IL-21R in BPH-1 cells, using the techniques of qRT-PCR and Western blot. Results showed no significant difference in IL-21R expression (Supplementary Figures S2, S3). These results suggest that THP-1 cells or LPS has no effect on IL-21R expression in BPH-1 cells. Therefore, AcTHP-1 cells were co-cultured with BPH-1 cells for follow-up experiments. As shown in Figure 6, co-culturing with AcTHP-1 cells *per se* heavily up-regulated IL-21R. Accordingly, flow cytometry showed cell apoptosis and cell cycle arrest induced by silencing IL-21R were reversed in co-cultured cells (Figures 7A–D). Consistently, the levels of proteins (BAX, Bcl-2, Cyclin D1, CDK4, and CDK6) involved in cell apoptosis and the cell cycle were reversed by co-culturing with AcTHP-1 (Figures 7E,F). Additionally, immunofluorescent staining and Western Blotting showed protein expressions of E-Cad, N-Cad, and vimentin associated with EMT process were also reversed with E-Cad decreased while N-Cad and vimentin increased (Figure 8). Again, our data indicated that IL-21R could modulate cell apoptosis and the cell cycle, as well as the EMT process, via an inflammatory microenvironment in the prostate.

**Figure 9:**
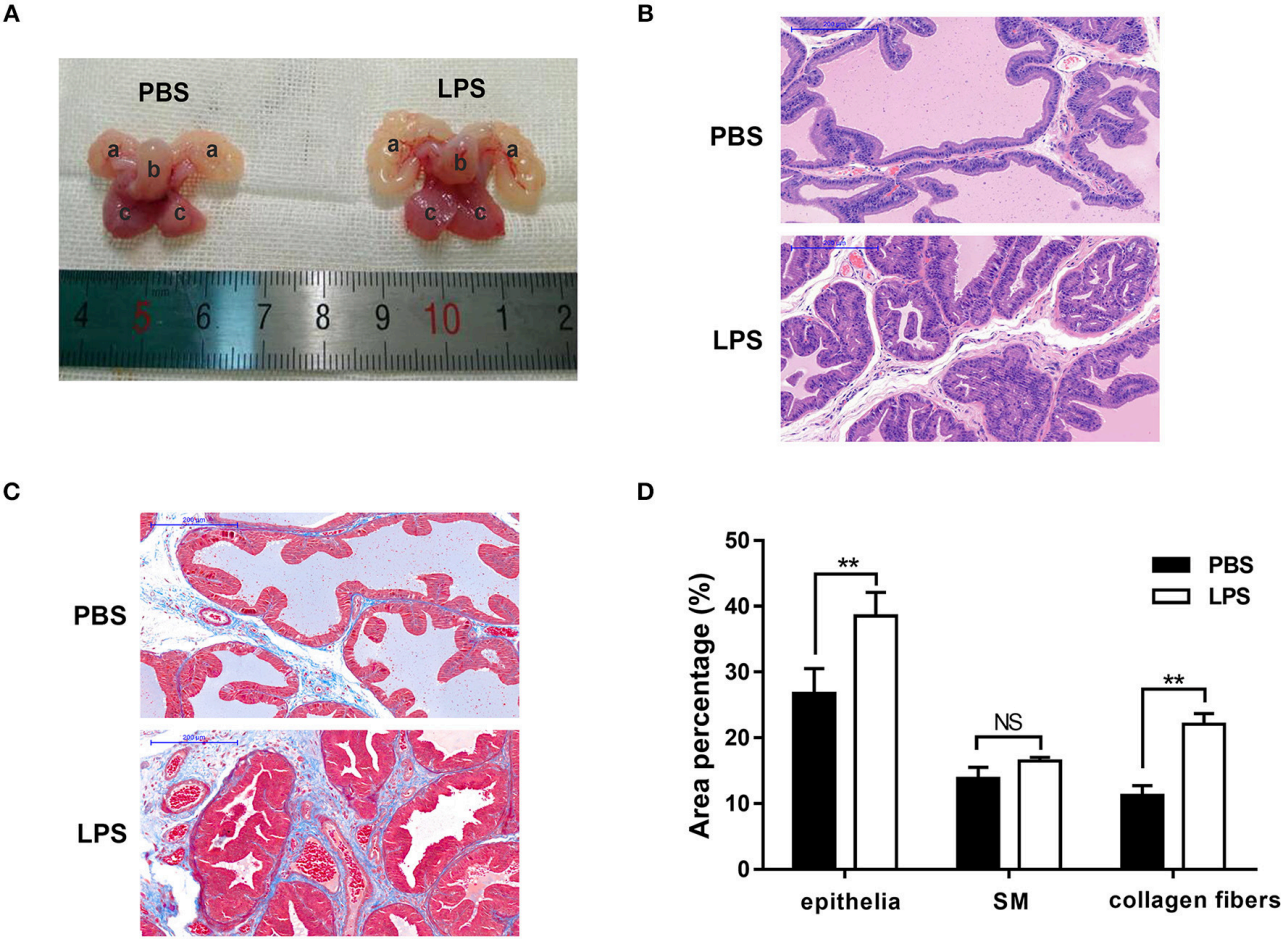
Typical rat prostate photograph and histological examination. **(A)** typical prostate photograph from PBS and LPS treatment rats, (a) seminal vesicle, (b) bladder, (c) prostate. **(B)** Representative H-E staining of PBS and LPS treatment rat prostate. The scale bars are 200 μm. **(C)** Masson's trichrome staining of PBS and LPS treatment rat prostate, prostate epithelial cells were stained orange, SM cells were stained red and collagen fibers were stained blue. The scale bars are 200 μm. (*n* = 8 for each group). **(D)** Quantification of Masson's trichrome staining. Area percentage of different component were quantified from three random 100 × fields of each tissue slices (*n* = 8 for each group). Boxes, mean; bars, ± SD; ^**^P < 0.01, NS means no significance vs. PBS.

**Figure 10:**
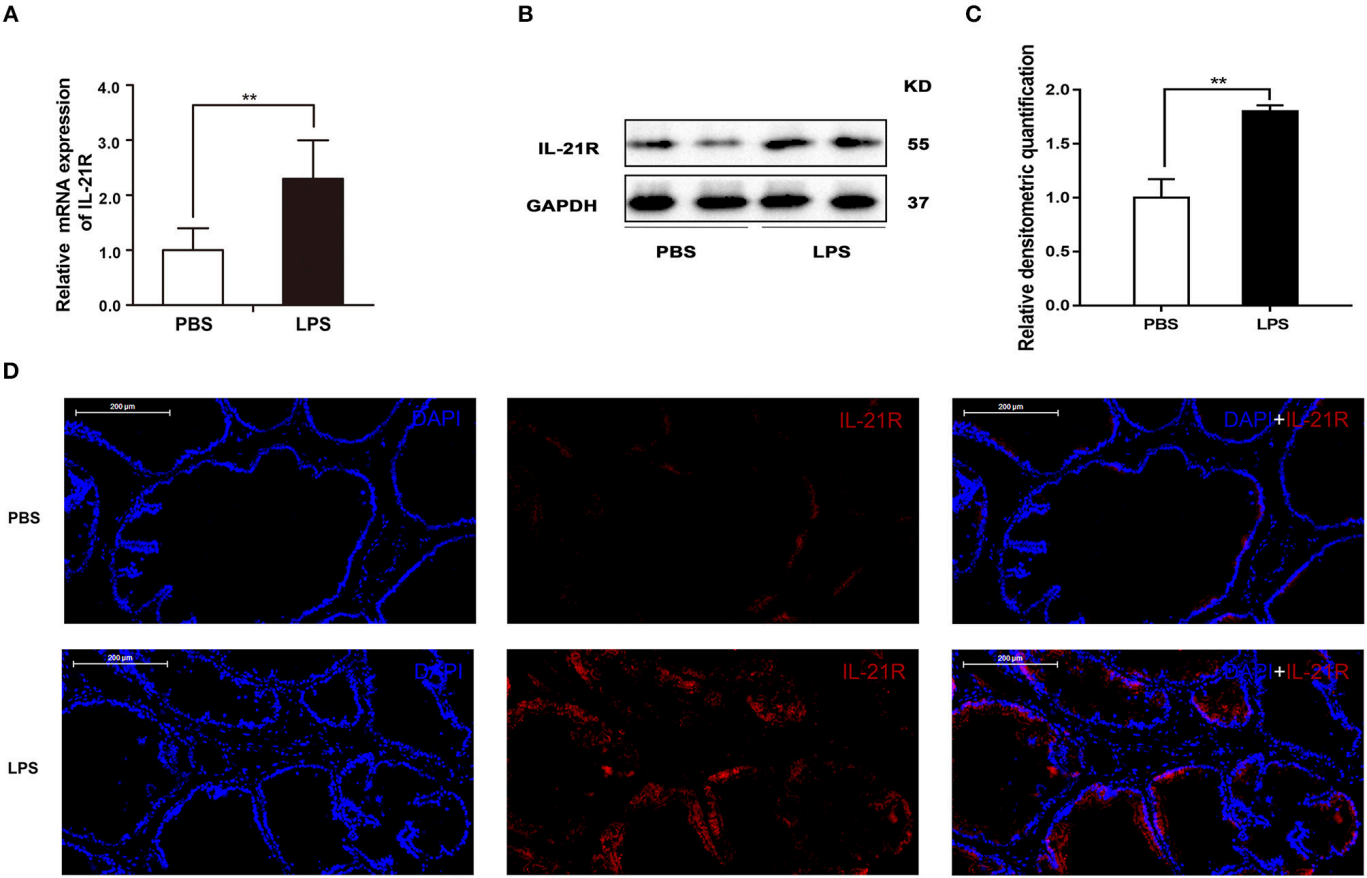
The expression and immunofluorescence of IL-21R in rat prostate. **(A)** The mRNA expression of IL-21R in PBS and LPS treated prostate (*n* = 8 for each group); **(B)** representative Western blot band of IL-21R in PBS and LPS treated rat prostate. **(C)** Relative densitometric quantification of IL-21R in PBS and LPS treated rat prostate. GAPDH expression was analyzed as a loading control, results are expressed as ratio of IL-21R in respect to GAPDH. Boxes, mean; bars, ± SD; ^**^P < 0.01 vs. PBS. **(D)** Immunofluorescence of IL-21R. Left, DAPI (blue) indicates nuclear staining. Middle, Cy3-immunofluorescence (red) indicates IL-21R. Right, Merged image. The scale bars are 200 μm.

**Figure 11:**
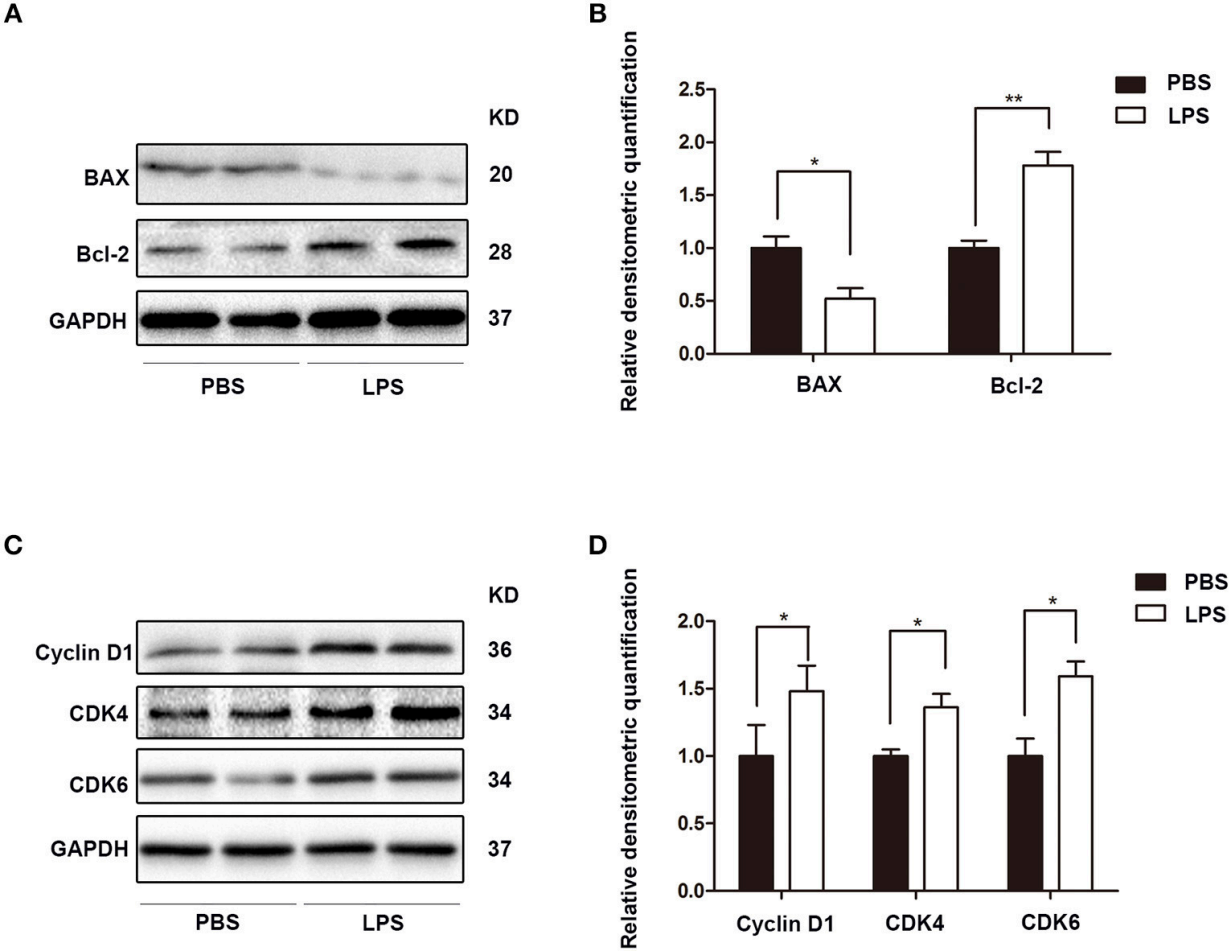
The expression of apoptosis and cyclin proteins in rat prostate. **(A)** Representative Western Blot band of BAX and Bcl-2 in PBS and LPS treatment rat prostate. **(B)** Relative densitometric quantification of BAX and Bcl-2 in PBS and LPS treatment rat prostate. GAPDH expression was analyzed as a loading control, results are expressed as ratio of the proteins in respect to GAPDH. Boxes, mean; bars, ± SD; ^*^P < 0.05 vs. LPS. **(C)** Representative Western Blot band of Cyclin D1, CDK4, and CDK6 in PBS and LPS treatment rat prostate. **(D)** Relative densitometric quantification of Cyclin D1, CDK4, and CDK6 in PBS and LPS treatment rat prostate. GAPDH expression was analyzed as a loading control, results are expressed as ratio of the proteins in respect to GAPDH. Boxes, mean; bars, ± SD; ^*^P < 0.05, ^**^P < 0.01 vs. PBS.

**Figure 12:**
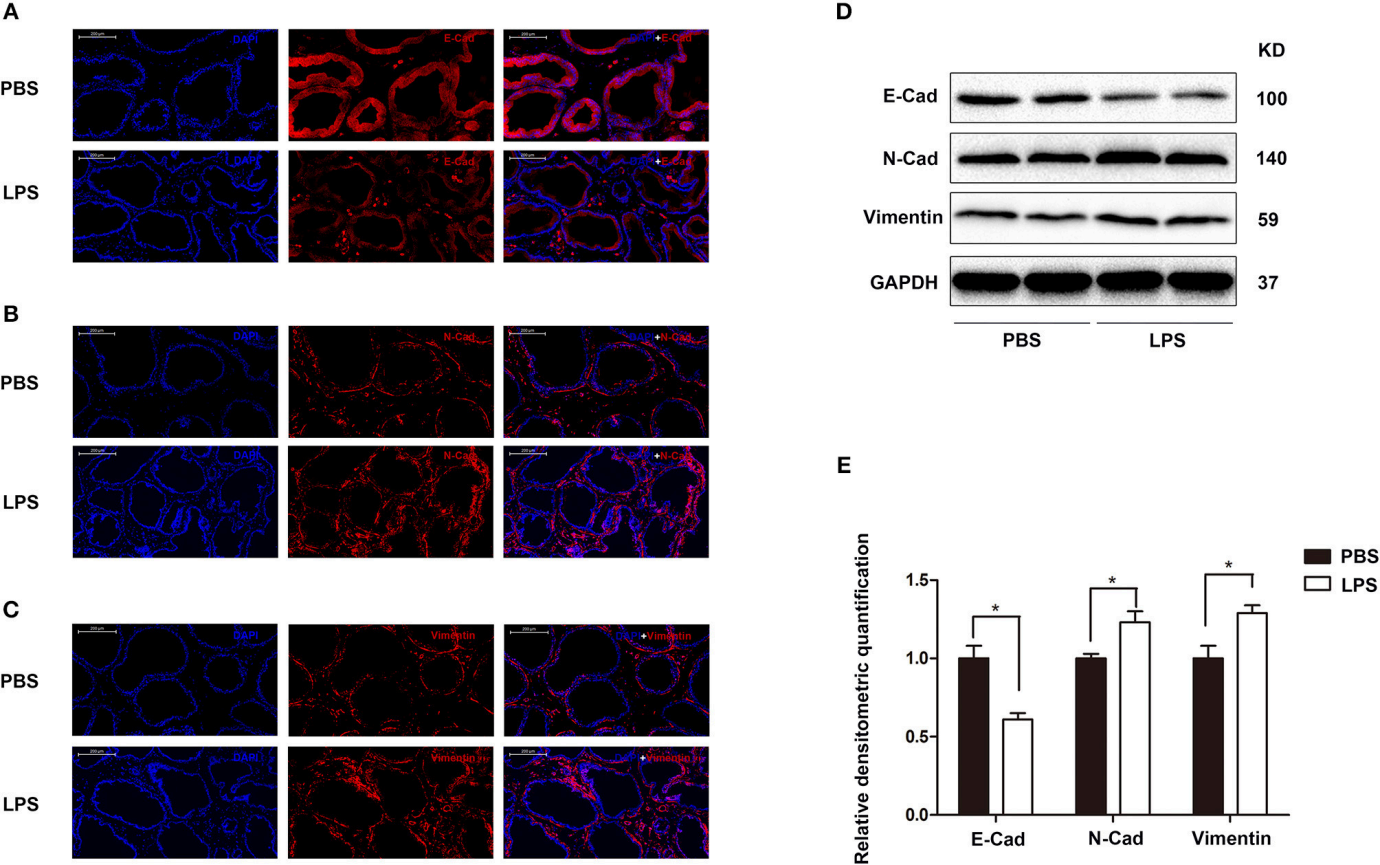
The expression and immunofluorescence of EMT proteins in rat prostate. **(A–C)** Immunofluorescence of E-Cad, N-Cad, and vimentin in PBS and LPS treatment rat prostate. Left, DAPI (blue) indicates cell nuclear staining. Middle, Cy3-immunofluorescence (red) indicates E-Cad **(A)**, N-Cad **(B)**, and vimentin **(C)** expression. Right, Merged image. The scale bars for **(A–C)** are 200 μm. **(D)** Representative Western Blot band of E-Cad, N-Cad, and vimentin in PBS and LPS treatment rat prostate. **(E)** Relative densitometric quantification of E-Cad, N-Cad, and vimentin in PBS and LPS treatment rat prostate. GAPDH expression was analyzed as a loading control, results are expressed as ratio of IL-21R in respect to GAPDH. Boxes, mean; bars, ± SD; ^*^P < 0.05 vs. PBS.

Finally, the role of IL-21R was determined *in vivo*. With LPS intraprostatic injection, a prostatitis rat model was established. This model exhibited inflammatory infiltration and hemorrhage (Figure 9B) shown by H & E Staining. Interestingly, our 2-week prostatitis rats were concomitant with prostatic hyperplasia. As shown in Figure 9A and Table 2, rat prostates were significantly enlarged with the weight of the ventral prostate increased by 1.9-fold and the prostate index [prostate wet weight (mg)/ body weight (g)] increased by 2.0-fold (*P* < 0.01) in the LPS injected rats group. No difference for the body weight was observed between the 2 groups (Table 2). In addition, it was observed the number of acini increased, lumen space decreased, epithelium thickened, and stroma densified (Figure 9B) in the LPS injected group as shown in H & E staining. Masson's trichrome stain in further showed the epithelium and the collagen fibers component increased with no change in the stroma (Figures 9C,D). Similar to human BPH, IL-21R was mainly localized in the epithelium of rat prostate (Figure 10D) and its expression was upregulated with the levels of mRNA (Figure 10A) and protein (Figures 10B,C) both enhanced in LPS injected group, when compared to normal rat prostate tissues. In addition, we detected apoptosis, cyclin and EMT proteins in this rat model by Western blot and immunofluorescent staining. As shown in Figures 11, 12, BAX was decreased while Bcl-2 was increased (Figures 11A,B). Cyclin D1, CDK4, and CDK6 were all significantly increased (Figures 11C,D). E-Cad was down-regulated, while N-Cad and vimentin were up-regulated (Figure 12). These results are consistent with that observed in the cell line model.

**Table 2:** Variation of biometric and physiological parameters in PBS and LPS injected rats.

	**Body weight (g)**			
**Group**	**Initial**	**Final**	**Ventral prostate weight (mg)**	**Prostate index**
PBS	270 (25)	315 (49)	250 (66.7)	0.79 (0.00)
LPS	268 (16)	319 (36)	470 (266.7)**	1.50 (0.01)**
*P*-value	0.62	0.49	< 0.01	< 0.01

*P-values calculated by unpaired t-test. Data are mean ± SD*.

** *P < 0.01 vs. PBS*.

## Discussion

Our novel data showed IL-21R was mainly localized in prostate epithelium and it was upregulated in hyperplastic prostate tissues. Our study also demonstrated that IL-21R plays roles in the development of BPH via inhibiting cell apoptosis and modulating cell cycle progression, as well as enhancing the EMT process in response to inflammatory stimuli.

IL-21R is primarily expressed in hematopoietic cells but it has also been found to exist in non-immune cells such as fibroblasts, endothelial cells, keratinocytes, Hodgkin lymphoma cells, and breast cancer cells (20, 22, 23). In this current study, we found that IL-21R was highly expressed in prostate tissues, especially in the epithelium. Moreover, it was upregulated in the prostates from BPH patients concomitant with prostatitis. Consistent with IL-21R, differential expression of other IL-receptors have been observed between BPH and normal prostate tissues, such as IL-15R (26). Additionally, previous studies have suggested IL-2, IL-4, and IL-15 are partly secreted by epithelial cells in response to inflammatory stimuli at the onset and during the progression of BPH (8, 13, 15). Although it was known that IL-21 is mainly released from T-lymphocytes, it has not been determined whether IL-21, similar to IL-2, IL-4, and IL-15, is also partly secreted by epithelial cells.

We further explored the mechanisms of IL-21R in the development of BPH. As IL-21R was mainly expressed in the epithelium, the cultured human prostate epithelium cell line BPH-1 was used. When the expression of IL-21R in BPH-1 was knocked down, the apoptosis rate was increased while the cell cycle was arrested, which were demonstrated by flow cytometry and related pathway proteins. In addition, the EMT process was inhibited when IL-21R was silenced. On the other hand, when the siIL-21R1 transfected cells were co-cultured with AcTHP-1 which could release cytokines, IL-21R expression was up-regulated and the aforementioned processes were *per se* reversed. Therefore, our data suggests that IL-21R possibly mediates prostatic cell apoptosis, cell cycle and the EMT process during the inflammatory microenvironment. Similarly, previous studies have shown that IL-21 attracts regulatory T-cells via up-regulation of macrophage inflammatory protein-3α and protects cells from apoptosis via activation of STAT3 signaling pathways in IL-21R^+^ Hodgkin lymphoma cells (29). A recent study also showed that IL-21 increased the proliferation of IL-21R^+^ MDA-231 breast cancer cells but not that of other breast cancer cells (20). EMT is regarded as a conserved cellular process that allows the polarized and generally immotile epithelial cells to convert to motile mesenchymal cells.

A number of studies have suggested that BPH development involves accumulation of mesenchymal-like cells derived from the prostatic epithelium via EMT (5–7). Given that BPH is perceived as an immune-mediated inflammatory disease, chronic inflammation may directly stimulate the development of BPH (8–10, 30) and macrophages are the major component of the prostate inflammatory infiltrates (16). A previous study has supposed macrophages secrete cytokines, such as TGFβ, to induce the proliferation and EMT process of BPH-1 cells via co-culture of BPH-1 cells with macrophages (31). LPS was established as a chemical inducer to differentiate THP-1 cells into macrophages (32). Therefore, LPS was used for differentiation of THP-1 cells in our study. Our study found THP-1 cells had no effect on IL-21R expression in BPH-1 cells. It is because THP-1 is a human leukemia monocytic cell line and these cells are often described as being in an activation state in *in vitro* experiments, which can be obtained by stimulating THP-1 cells with inflammatory activators, for instance, LPS, or pro-inflammatory cytokines (33–35). Our data is consistent with the previous study that macrophages, which differentiate from THP-1 cells, induced EMT in BPH development (31). Moreover, IL-21R expression was not changed in BPH-1 cells which were stimulated with a gradient concentration of LPS. It is also consistent with the previous study that LPS has no direct effect on prostate cells (36). Indeed, the current study found macrophages reversed the silencing of IL-21R related processes when co-culture was performed. However, the exact cytokines binding to IL-21R and the underling pathways of activating IL-21R in the inflammatory environment, although intriguing, remain to be elucidated.

We further extended our study *in vivo*. We injected LPS into rat prostates for 14 days. LPS did indeed produce prostatitis and concomitant prostatic hyperplasia. Consistent with human BPH and our *in vitro* cell proliferation study, IL-21R was upregulated in the prostate of the LPS induced prostatitis and BPH rat model. More specifically, the expression of apoptosis, cyclin, and EMT proteins in this rat model are altered in a manner consistent with that seen in the cell line model. It has been determined that LPS activates the immune system through stimulating pro-inflammatory cytokines and growth factors (37, 38). In addition, it has been suggested that LPS/TLR4 (Toll-like receptor 4) signaling enhances the TGF-β response during prostatic hyperplasia (36). Similarly, the activation of the expression of cytokines and growth factors was observed in prostatitis and BPH model induced with LPS injected into the rat prostate and mouse urethra (17, 39). Previous studies also showed that there was co-localization of IL-21R and VEGF in the keratinocytes of patients with skin sclerosis and it was demonstrated that VEGF production was modulated by IL-21 through IL-21R (40, 41), suggesting IL-21R is associated with inflammation and proliferation. However, it is premature to attribute the observed effects on the differential expression of IL-21R. It may be that IL-21R upregulation is a marker of chronic inflammation/BPH downstream of other factors central to the development of the pathology. Therefore, the effect of LPS injection on prostate biology in IL-21R knockout mice is of great interest for future investigation.

In summary, this is the first study to demonstrate the expression and functional activities of IL-21R in the prostate. IL-21R is mainly localized in prostate epithelium and it is upregulated in hyperplastic prostate tissues. Moreover, IL-21R appears to be involved in the development of BPH via modulation of cell apoptosis and cell cycle progression, as well as the EMT process in the inflammatory microenvironment. Thus, our data suggests that IL-21R could be a promsing new therapeutic target for the treatment of BPH.

## Author Contributions

DX, PC, and HX contributed equally to this work. XZ, DX, PC, XW, and MD designed the experiments. DX and HX finished the experiments. DX wrote the first draft. DX and PC analyzed the results. XZ critically revised drafts of the manuscript. XZ provided important intellectual input and approved the final version for publication.

### Conflict of Interest Statement

The authors declare that the research was conducted in the absence of any commercial or financial relationships that could be construed as a potential conflict of interest.

## Supplementary Material

Supplementary Figure S1 The expression of IL-21R in prostate cells. **(A)** The mRNA expression of IL-21R in BPH-1 and WPMY-1 cells. **(B)** Representative Western Blot band of IL-21R in BPH-1 and WPMY-1 cells. **(C)** Relative densitometric quantification of IL-21R in BPH-1 and WPMY-1 cells. GAPDH expression was analyzed as a loading control, results are expressed as ratio of IL-21R in respect to GAPDH. Boxes, mean; bars, ± SD; ^**^*P* < 0.01 vs. BPH-1.

Supplementary Figure S2 Effect of THP-1 co-culture on the expression of IL-21R in BPH-1 cells. **(A)** The mRNA expression of IL-21R in BPH-1 cells co-cultured with or without THP-1cells. **(B)** Representative Western Blot bands of IL-21R in BPH-1 cells co-cultured with or without AcTHP-1cells. **(C)** Relative densitometric quantification of IL-21R in BPH-1 cells. GAPDH expression was analyzed as a loading control, results are expressed as ratio of the proteins in respect to GAPDH. Boxes, mean; bars, ± SD; NS means no significance, BPH-1cells without THP-1 co-culture vs. BPH-1cells with THP-1 co-culture.

Supplementary Figure S3 Effect of LPS on the mRNA expression of IL-21R in BPH-1 cells. The mRNA expression of IL-21R in BPH-1 cells treated with gradient concentration of LPS. Boxes, mean; bars, ± SD; NS means no significance vs. control.

Supplementary Table S1 List of siRNA sequences.

Supplementary Table S2 List of primary antibodies used for western blot.

Supplementary Table S3 List of secondary antibodies used for western blot.

## Abbreviations

BPH: benign prostatic hyperplasia
ILs: interleukins
IL-2R: interleukin 2 receptor
IL-21R: interleukin 21 receptor
AcTHP-1: active THP-1
EMT: epithelial-mesenchymal transition
LPS: lipopolysaccharide
H & E: Hematoxylin and Eosin
JAK/STAT: Janus-faced kinase/signal transducer and activator of transcription
qRT-PCR: quantitative real-time PCR
SM: smooth muscle
E-Cad: E-cadherin
N-Cad: N-cadherin
SD: standard deviation.
