# Supplementary figures and images for: Upregulated Interleukin 21 Receptor Enhances Proliferation and Epithelial-Mesenchymal Transition Process in Benign Prostatic Hyperplasia
Source: Front Endocrinol (Lausanne). 2019 Jan 23;10:4. doi: 10.3389/fendo.2019.00004 (PMC6351785; doi:10.3389/fendo.2019.00004)

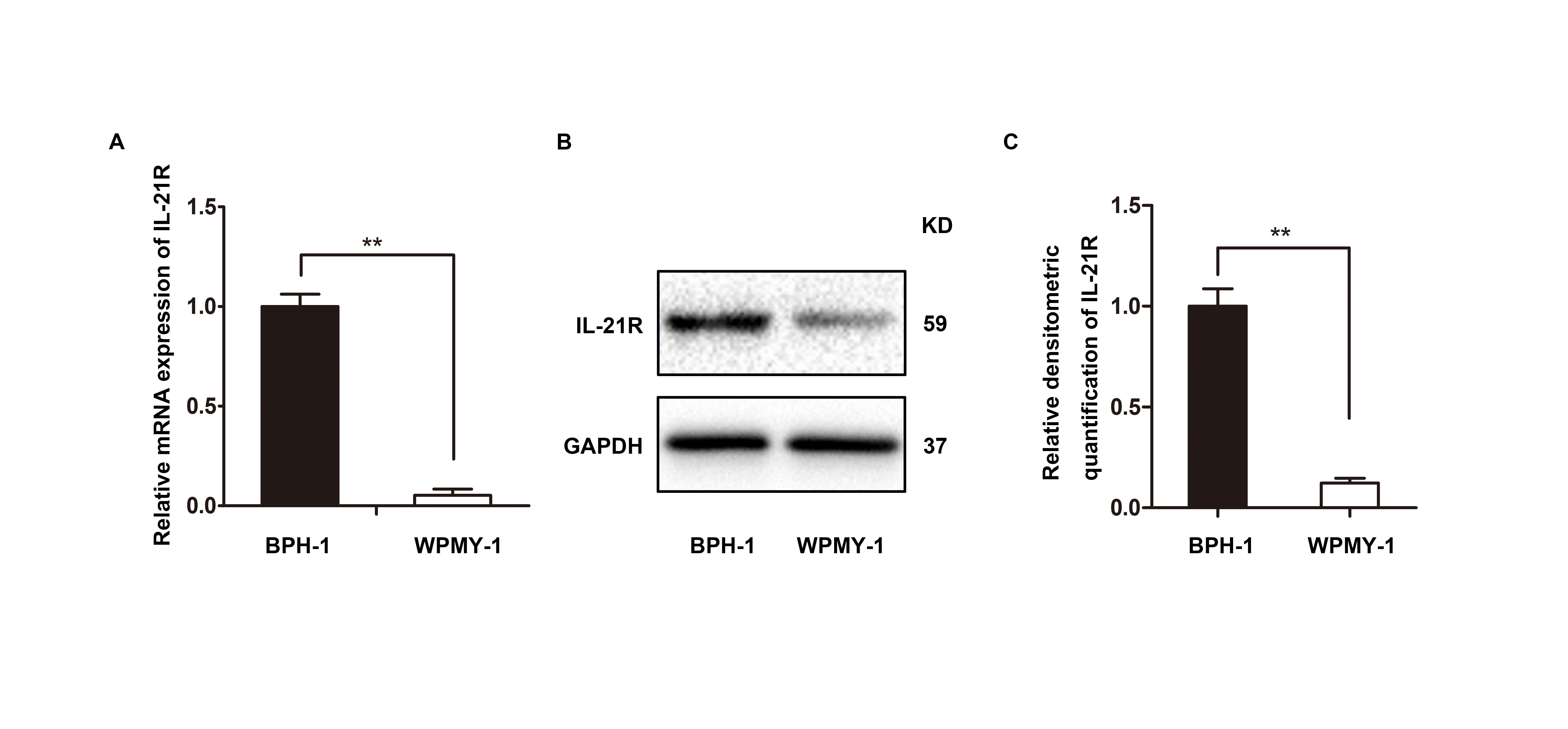

Supplement: Supplementary Figure S1 — The expression of IL-21R in prostate cells. (A) The mRNA expression of IL-21R in BPH-1 and WPMY-1 cells. (B) Representative Western Blot band of IL-21R in BPH-1 and WPMY-1 cells. (C) Relative densitometric quantification of IL-21R in BPH-1 and WPMY-1 cells. GAPDH expression was analyzed as a loading control, results are expressed as ratio of IL-21R in respect to GAPDH. Boxes, mean; bars, ± SD; **P < 0.01 vs. BPH-1. [file Image_1.tif]

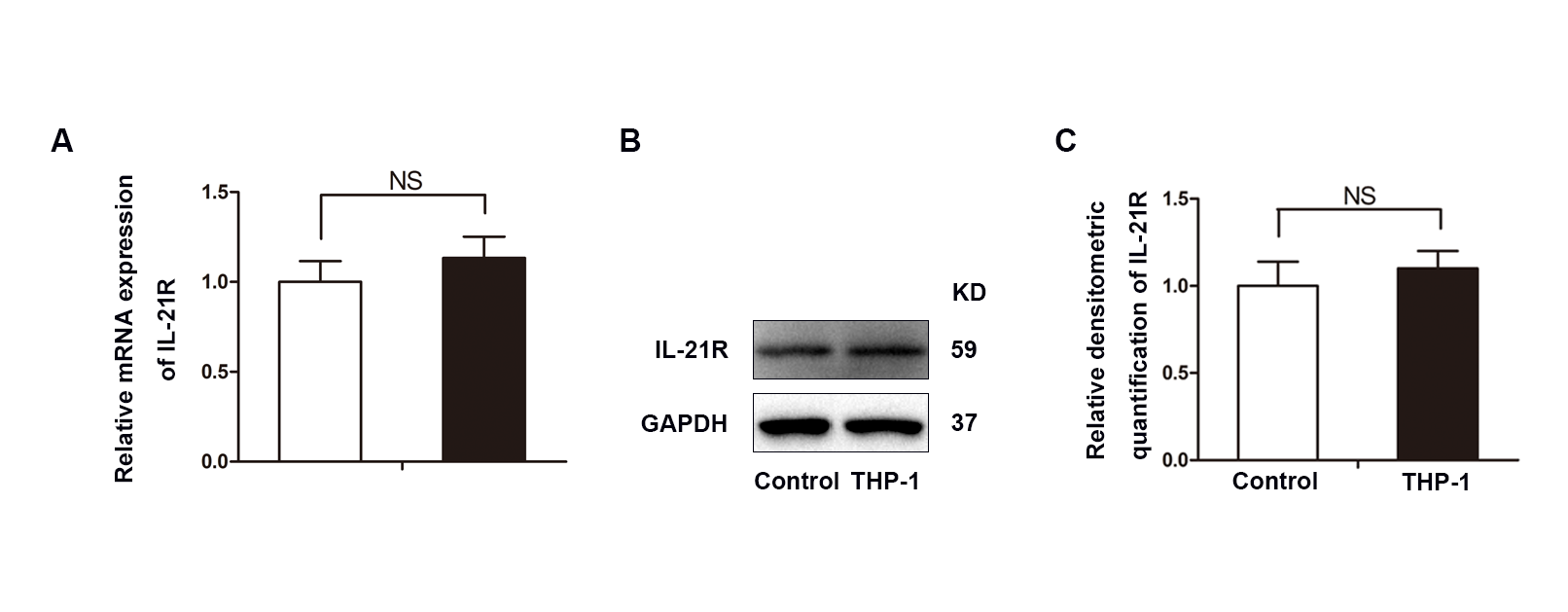

Supplement: Supplementary Figure S2 — Effect of THP-1 co-culture on the expression of IL-21R in BPH-1 cells. (A) The mRNA expression of IL-21R in BPH-1 cells co-cultured with or without THP-1cells. (B) Representative Western Blot bands of IL-21R in BPH-1 cells co-cultured with or without AcTHP-1cells. (C) Relative densitometric quantification of IL-21R in BPH-1 cells. GAPDH expression was analyzed as a loading control, results are expressed as ratio of the proteins in respect to GAPDH. Boxes, mean; bars, ± SD; NS means no significance, BPH-1cells without THP-1 co-culture vs. BPH-1cells with THP-1 co-culture. [file Image_2.tif]

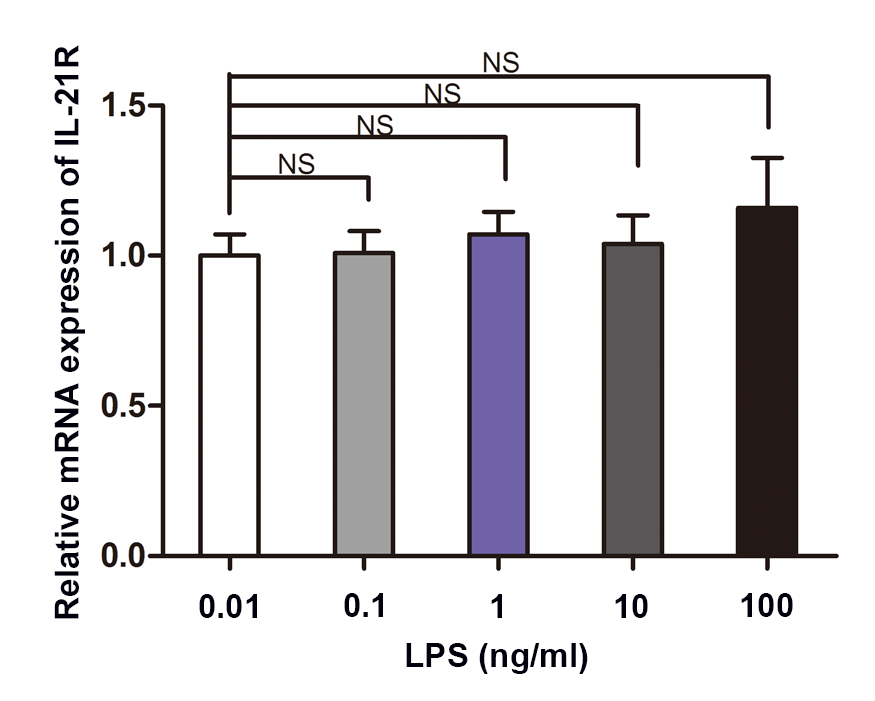

Supplement: Supplementary Figure S3 — Effect of LPS on the mRNA expression of IL-21R in BPH-1 cells. The mRNA expression of IL-21R in BPH-1 cells treated with gradient concentration of LPS. Boxes, mean; bars, ± SD; NS means no significance vs. control. [file Image_3.tif]
