# Supplementary material for: Upregulated Interleukin 21 Receptor Enhances Proliferation and Epithelial-Mesenchymal Transition Process in Benign Prostatic Hyperplasia
Source: Front Endocrinol (Lausanne). 2019 Jan 23;10:4. doi: 10.3389/fendo.2019.00004 (PMC6351785; doi:10.3389/fendo.2019.00004)
Supplement: Supplementary Table S1 — List of siRNA sequences. [file Table_1.docx]

Supplementary Table S1. List of siRNA sequences.

| Symbol | Sense sequences | Antisense sequences |
| --- | --- | --- |
| SiIL-21R1 | 5’-CCUGCCACAUGGAUGUAUUTT-3’ | 5’-AAUACAUCCAUGUGGCAGGTT-3’ |
| SiIL-21R2 | 5’-CCGCAAAGACUCGAGCUAUTT-3’ | 5’-AUAGCUCGAGUCUUUGCGGTT-3’ |
| SiIL-21R3 | 5’-CCAUCCAUUGUGGAGGCUATT-3’ | 5’-UAGCCUCCACAAUGGAUGGTT-3’ |
| Control siRNA (sicon) | 5’-UUCUCCGAACGUGUCAGGUTT-3’ | 5’-ACGUGACACGUUCGGAGAATT-3’ |
