# Supplementary material for: Upregulated Interleukin 21 Receptor Enhances Proliferation and Epithelial-Mesenchymal Transition Process in Benign Prostatic Hyperplasia
Source: Front Endocrinol (Lausanne). 2019 Jan 23;10:4. doi: 10.3389/fendo.2019.00004 (PMC6351785; doi:10.3389/fendo.2019.00004)
Supplement: Supplementary Table S2 — List of primary antibodies used for western blot. [file Table_2.docx]

Supplementary Table S2. List of primary antibodies used for western blot.

| Antigens | Species Antibodies Raised in | Dilution | Supplier |
| --- | --- | --- | --- |
| IL-21R | Rabbit monoclonal | 1:500 | Abcam |
| BAX | Rabbit monoclonal | 1:1000 | Cell Signaling Technology |
| Bcl-2 | Rabbit monoclonal | 1:2000 | Cell Signaling Technology |
| CDK4 | Rabbit monoclonal | 1:2000 | Cell Signaling Technology, Cat. #12790 |
| CDK6 | Rabbit, monoclonal | 1:1000 | Abcam, Cat. #ab124821 |
| Cyclin D1 | Rabbit, monoclonal | 1:2000 | Cell Signaling Technology, Cat. #2978 |
| E-Cad | Rabbit, monoclonal | 1:500 | Cell Signaling Technology, Cat. #3195 |
| N-Cad | Rabbit, monoclonal | 1:1000 | Cell Signaling Technology, Cat. #13116 |
| Vimentin | Rabbit, monoclonal | 1:2000 | Cell Signaling Technology, Cat. #5741 |
