# Supplementary material for: Upregulated Interleukin 21 Receptor Enhances Proliferation and Epithelial-Mesenchymal Transition Process in Benign Prostatic Hyperplasia
Source: Front Endocrinol (Lausanne). 2019 Jan 23;10:4. doi: 10.3389/fendo.2019.00004 (PMC6351785; doi:10.3389/fendo.2019.00004)
Supplement: Supplementary Table S3 — List of secondary antibodies used for western blot. [file Table_3.docx]

Supplementary Table S3. List of secondary antibodies used for western blot.

| Secondary Detection System Used | Host | Dilution | Supplier |
| --- | --- | --- | --- |
| Anti-Mouse-IgG (H + L)-HRP | Goat | 1:10,000 | Sungene Biotech, Tianjin, China, Cat. #LK2003 |
| Anti-Rabbit-IgG (H + L)-HRP | Goat | 1:10,000 | Sungene Biotech, Cat. #LK2001 |
